# QTL Alignment for Seed Yield and Yield Related Traits in *Brassica napus*

**DOI:** 10.3389/fpls.2018.01127

**Published:** 2018-08-02

**Authors:** Nadia Raboanatahiry, Hongbo Chao, Hou Dalin, Shi Pu, Wei Yan, Longjiang Yu, Baoshan Wang, Maoteng Li

**Affiliations:** ^1^Department of Biotechnology, College of Life Science and Technology, Huazhong University of Science and Technology, Wuhan, China; ^2^Hubei Key Laboratory of Economic Forest Germplasm Improvement and Resources Comprehensive Utilization, Hubei Collaborative Innovation Center for the Characteristic Resources Exploitation of Dabie Mountains, Huanggang Normal University, Huanggang, China; ^3^College of Life Sciences, Shandong Normal University, Jinan, China

**Keywords:** *Brassica napus*, quantitative trait loci, alignment map, seed-yield, yield-related traits, candidate genes

## Abstract

Worldwide consumption of oil is increasing with the growing population in need for edible oil and the expansion of industry using biofuels. Then, demand for high yielding varieties of oil crops is always increasing. *Brassica napus* (rapeseed) is one of the most important oil crop in the world, therefore, increasing rapeseed yield through breeding is inevitable in order to cater for the high demand of vegetable oil and high-quality protein for live stocks. Quantitative trait loci (QTL) analysis is a powerful tool to identify important loci and which is also valuable for molecular marker assisted breeding. Seed-yield (SY) is a complex trait that is controlled by multiple loci and is affected directly by seed weight, seeds per silique and silique number. Some yield-related traits, such as plant height, biomass yield, flowering time, and so on, also affect the SY indirectly. This study reports the assembly of QTLs identified for seed-yield and yield-related traits in rapeseed, in one unique map. A total of 972 QTLs for seed-yield and yield-related were aligned into the physical map of *B. napus Darmor-bzh* and 92 regions where 198 QTLs overlapped, could be discovered on 16 chromosomes. Also, 147 potential candidate genes were discovered in 65 regions where 131 QTLs overlapped, and might affect nine different traits. At the end, interaction network of candidate genes was studied, and showed nine genes that could highly interact with the other genes, and might have more influence on them. The present results would be helpful to develop molecular markers for yield associated traits and could be used for breeding improvement in *B. napus*.

## Introduction

Rapeseed (*Brassica napus*) is an important crop utilized globally for vegetable oil production, animal feed and biofuel. *B. napus* (AACC, 2n = 38) is a polyploid species, which was derived from the hybridization between turnip rape (*B. rapa*, AA, 2n = 20) and cabbage (*B. oleracea*, CC, 2n = 18) (Nagaharu, 1935; Al-Shehbaz et al., 2006; Bailey et al., 2006; Chalhoub et al., 2014). In terms of evolutionary age of plants in *Brassica* genera, *B. napus* registers the shortest age and history of domestication (Jesske et al., 2013; Chalhoub et al., 2014). Spring type *B. napus* are typically cultivated in Canada, Asia, Eastern Europe, and Australia, whereas winter type *B. napus* are usually grown in Western Europe; those winter type *B. napus* generally gives higher SYs than spring type (Nesi et al., 2008). Currently, the most important task for plant breeders is to develop a high yielding variety in order to attain sustainable production in future. Rapeseed yield (SY per plant) is determined by the number of siliques per plant, the number of seeds per silique and the SW. The total number of seeds are determined by the number of seeds per siliques and the number of siliques per plant (Chen et al., 2007).

Genetic approach has become the most powerful tool for examining the genetic traits in many crops including *B. napus*. Application of molecular marker techniques for quantitative trait loci (QTL) is a trending strategy (Paran and Zamir, 2003). The major objective of QTL studies is to find genes that could be used in breeding via marker assisted selection (MAS). QTL mapping is also a potent method which is widely used to decipher regulatory loci and genetic mechanism of traits. Identification of QTLs is necessitated by molecular markers coupled with mapping populations. The magnitude and presence of QTLs varies between experiments, so the sensitivity of the QTL can easily be underscored. Also, the number of QTLs identified in different populations varies with the genetic background of parental lines, the type and size of the population, and the number of environments that were used in different studies (Zhu and Zhao, 2007).

Seed yield is a very complex trait. In an open-pollinated and hybrid type crop, such as rapeseed, high SY is partly attributed to epistatic interaction between the loci and possible multiple alleles at the loci which control SY trait (Engqvist and Becker, 1991; Radoev et al., 2008). Moreover, the effects of the QTL alleles increasing SY on a particular genetic background could produce a different phenotypic effect on another genetic background, and the alleles may be responsible for very poor yield in some cases (Kramer et al., 2009). Therefore, the fact that QTL alleles may exhibit an effect in a certain combination of alleles that involves one or several loci, and another different effect in another set of allele combinations might suffice the need for researchers to discover and determine the effects of different genes in varying environment. This will enable breeding programs to be effective, since the different individual alleles from each locus, as well as allelic combinations from different loci, will be utilized in identification of the most favorable gene combinations that will produce maximum benefit. Nevertheless, this task is not simple considering that more than one QTL regions and multiple variants of alleles at every locus have the ability to control quantitative traits, such as SY and yield associated traits. Also, as mentioned earlier, it is clear that QTL vary with genetic × environment background (Zhu and Zhao, 2007), therefore, combination of multiple QTLs from different genetic × environmental background into one map would allow an easy comparison between these QTLs. Establishing a consensus map allows comparison between these multiple QTLs and valuable findings might be obtained. For example, Wang et al. (2013) constructed a consensus map of 2395.2 cM genome length with 1335 markers to compare QTL for oil content in seven different populations of *B. napus*. They affirmed that combining multiple QTLs from different population into one consensus map could be a potential tool not only to study the genetic diversity of loci with complex traits, but also to develop molecular breeding and map-based cloning of genes (Wang et al., 2013). Additionally, Zhou et al. (2014) performed *in sillico* integration of QTLs for SY and yield related trait from 15 *B. napus* mapping populations by using *B. rapa* and *B. oleracea* genome sequences, and 736 SY and yield related QTLs on 283 loci of *B. napus* A and C genome were mapped, they were distributed across 19 chromosomes and most of them were located on A03. Also, Zhao et al. (2016) built a consensus map to compare QTLs for SY and yield related traits from five different populations of *B. napus*, 34 SY-QTLs could be projected onto the “*KenC-8 × N53-2*” map, half of them were placed on chromosome A02, A05, A06, C02, and C06. Nevertheless, difference in markers used with these QTLs makes difficult this combination into one consensus map and their comparison (Raman et al., 2013). Therefore, a QTL alignment map would help to overcome this difficulty. In fact, a QTL alignment map combines diverse QTLs from different genetic and environmental background. Therefore, QTLs from different populations which were developed in the same environments and which could overlap in one region might be defined as fixed QTLs for that environment, and QTLs that could not overlap with other QTLs might be specified QTLs. In this situation, fixed QTLs might be more stable and more important in further fine-mapping for gene cloning: firstly, the variation underlying the fixed QTL was more common in different varieties. If this variation is rare, it is impossible to detect it in many populations. Secondly, it can be detected in many populations and detected in different planting environments where each population can adapt to, which indicated that it is an ubiquitous variation (derived from early evolution) and has strong environment stability. Thus, conservative and variant regions could be uncovered with this QTL alignment map. Such technique was earlier used to compare multiple QTLs for seed oil content and fatty acid profile using *B. napus Darmor-bzh* as reference genome (Liu et al., 2016; Chao et al., 2017; Raboanatahiry et al., 2017). To date, establishment of QTL alignment map for SY and yield related traits has not been performed yet.

Identification of candidate genes implies the detection of important genes for agricultural and economic quantitative traits. Candidate genes are present within QTL region and they are responsible for phenotype variation. The effect of these genes on variation of phenotype could be elucidated through investigation on the gene arrangement and interaction of loci affecting this variation (Zhu and Zhao, 2007). Moreover, study on QTLs and related candidate genes could help in knowledge of their control on the phenotype (Remington and Purugganan, 2003; Zhu and Zhao, 2007). In *B. napus*, several studies on candidate genes for yield related traits have been performed before: for instance, Zhao et al. (2016) found four, two and one candidate genes for SY, SW and PH, respectively in a study performed with a double-halpoid (DH) population of 348 lines derived from a cross between *KenC-8 x N53-2.* Also, in a study executed with 333 diverse rapeseed accessions (20 winter type, 308 semi-winter type and 5 unknown accessions), candidate genes for PH (31 genes), branch initiation height (15 genes), and BN (17 genes) were found (Zheng et al., 2017). More, candidate genes for SN (six genes), PN per plant (seven genes), branch PN (seven genes), branch yield (three genes) and thousand seed weight (one gene) were discovered from a study made with 520 rapeseed accessions (Lu et al., 2017). In the model plant *Arabidopsis thaliana*, 425 genes were found to be related to BN, FT, MT, PH, PN, seed number, seed weight, and SY (Shi et al., 2009). In *B. napus*, homologous of these 425 genes of *A. thaliana* have not been searched before.

Therefore, the present study aimed to compare yield and yield-related QTLs in 22 populations which were developed in four different countries (**Table 1**) via arrangement into the physical map of *B. napus* “*Darmor-bzh*”, the candidate genes for SY and yield-related traits in *B. napus* were also searched inside the overlapping QTLs regions and a genetic network was constructed to understand the interaction of candidate genes.

**Table 1:** List of populations used for the alignment map.

Populations	Known relatives	Lines	Environments	Traits	Reference
*04-1139 × 05-1054*		221 F2	China	PN, SN	Wang and Guan, 2010
*448 lines*		448 IL	China	FT	Wang N. et al., 2016
*8008 x 4942C-5*	*8008 (Eyou Long Pod)*	181 DH	China	SN	Qi et al., 2014
*Express x SWU07*		261 DH/	China	SL, SW	Fu et al., 2015
		233 RC-F2			
*Express617 × R53*	*Express617 (Express)*	250 DH	Germany	PH, SW, SY, SPUA	Basunanda et al., 2010
	*Express617 (Express)*	250 DH	Germany	SY, SN, SPUA, SW	Radoev et al., 2008
*Express617 × V8*	*Express617 (Express)*	250 DH	Germany	PH, SPUA, SN, SW, SY	Basunanda et al., 2010
*HZ396 × Y106*		140 DH	China	SL, SW	Zhang et al., 2011
		807 DH	China	SN	Zhang et al., 2012
*J7046 × J7005*		190 F2	China	SW	Fan et al., 2010
*KenC-8 x N53-2*	*N53-2 (Midas, SE8)*	348 DH	China	SD, SL, SN, ST, SV	Wang X. et al., 2016
	*N53-2 (Midas, SE8)*	348 DH	China	BH, BY, FE BN, LMI, PH, PNMI, SW, SY	Zhao et al., 2016
*MF216 × P1804*	*MF216 (Stellar, Major)*	150 DH	America	FT, PH, SY, TW	Quijada et al., 2006
*MFDH × P124*		160 DH	America	FT, PH, SW, SY, TW	Quijada et al., 2006
*Quantum × NO.2127-17*	*NO. 2127-17, (resynthesized B. napus line NO. 7076)*	258 DH	China	NPB, PH, SID, SL	Chen et al., 2007
*Regent x Lagoda*		93 F2	Canada	FT, NRV	ArifUzZaman et al., 2016
*RV128 × P1804*	*RV128 (BC2S2, Westar, Samourai, Bienvenu, Major)*	150 DH	America	FT, PH, SW, SY, TW	Quijada et al., 2006
*RV289 × P1804*	*RV289 (Hua-dbl2)*	148 DH	America	PH, SW, SY, TW, FT	Udall et al., 2006
*RVDH × P124*		160 DH	America	FT, PH, SW, SY, TW	Quijada et al., 2006
*S1 × S2*	*S1 (breeding line 92-B10)*	186 RIL	China	SL, SW	Yang et al., 2012
*Sollux × Gaoyou*		282 DH	China, Germany	FT, MT, PH	Zhao et al., 2005
*Tapidor x Ningyou7*		182 DH	China	BN, BY, DTS, FBH, FT, MT, PH, PN, SN, SW, SY	Luo et al., 2017
		202 DH	China	NPB, BY, FT, MT, PH, PN, SN, SW, SY	Shi et al., 2009
		202 DH/	China	NPB, BY, FT, PH, MT, PN, PY, SN, SW, SY	Shi et al., 2011
		101 F2			
*TO1141 × P1804*	*TO1141 (B. rapa cv. Reward and B. oleracea TO1000)*	160 DH	America	FT, SL, PH, SW, SY, TW	Udall et al., 2006
*Westar(Marnoo) × Ceres*		128 IBL	America	PH, SW, FT	Butruille et al., 1999
*Zhongshuang11 x No.73290*		182 F2	China	SN	Shi et al., 2015
		184 RIL	China	SN	Yang et al., 2016
		184 F2/	China	PN	Ye et al., 2017
		184 RIL			


## Materials and Methods

### QTL Alignment Map for Seed-Yield and Yield Related Traits in *B. napus*

Quantitative trait loci for SY and yield related traits obtained from 22 populations were aligned to the physical map of *B. napus* winter type *Darmor-bzh*. To perform this alignment, QTL flanking markers were positioned on the map by using of E-PCR (Schuler, 1997; Rotmistrovsky et al., 2004). One marker could be detected on the genome owing to corresponding primer. So, primer sequences were searched on *Darmor-bzh* to spot the markers. Because some flanking marker sequences could not be found in some literatures or they are found but could not be placed on the map (misplaced to another chromosome or location not found on *Darmor-bzh*), some QTLs could not be aligned on the map. Also, it happened that only one flanking marker could be placed, and some literatures gave only one flanking marker; therefore, we took a uniform area of 1 cM to delimit all the QTLs, so that QTLs region could be located on the map even with one marker. Note that apart from related literatures, marker sequences could also be found on some database, such as Brassica.info^1^, NCBI^2^, Brassica IGF Project^3^, Gramene Markers Database^4^. The map was build using Circos software (Krzywinski et al., 2009).

### Candidate Genes Identification

As mentioned above, Shi et al. (2009) found 425 genes in *A. thaliana* which were related to BN, FT, MT, PH, PN, seed number, seed weight, and SY in their study; so in the current study, candidate genes were identified based on this study performed by Shi et al. (2009) First, genes related to yield and yield related traits in *B. napus* were identified based on homology to these 425 yield related genes in *A. thaliana*, by using Browse Data tool in http://www.genoscope.cns.fr/brassicanapus/. Then, we aligned these *B. napus* genes with the previous QTL alignment map of the current study. Consequently, candidate genes were genes located inside a QTL region.

Rapeseed genome showing beneficial stable loci with related candidate genes was then proposed. For that, overlapping QTLs identified previously, with candidate genes found inside the region of these overlapping QTLs were highlighted. A simple construction was made using an excel based tool called E-maps (Ulitsky et al., 2008).

### Construction of Candidate Genes Interaction Network

A network was constructed to understand the interaction between candidate genes. Interaction of proteins was predicted using STRING^5^ (Szklarczyk et al., 2015), clustered using Mapman software (Thimm et al., 2004) and then visualized with Cytoscape_V3.6.0 (Shannon et al., 2003). Orthologous genes in *A. thaliana* were used to perform the analysis, because *B. napus* is still not available on STRING database.

## Results

### Overlapping QTLs for Seed Yield and Yield-Related Traits Were Found Aligned in Physical Map of *Darmor-bzh*

**FIGURE 1:**
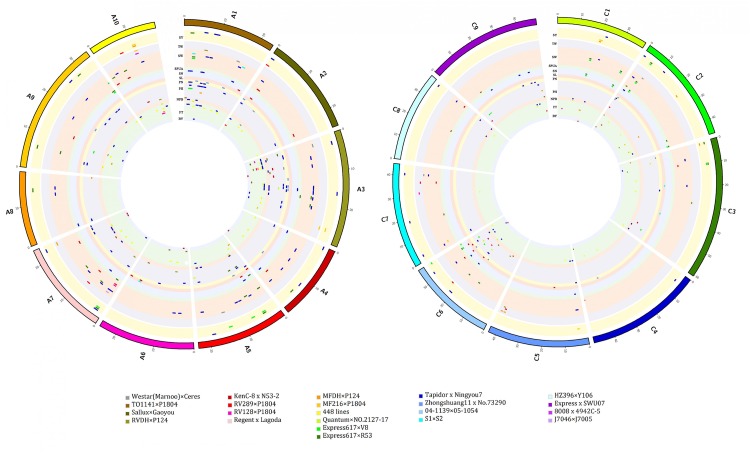
Alignment map displaying regions of overlapping QTLs for seed yield and yield-related traits in *B. napus*. From inside to outside, 11 inner circles with background color represent 11 traits (BY, FT, NPB, PH, PN, SL, SN, SPUA, SW, TW, and SY, respectively), and short bars with color within the 11 inner circles represent QTLs identified in different populations (represented on the left) and linkage groups. The blocks at the outermost circle represent the genetic linkage groups (A genome is on the left and C genome is on the right).

From the 22 populations, 972 QTLs for 26 yield traits could be aligned onto the physical map of *B. napus Darmor-bzh* (Supplementary Table 1): 182 (FT), 168 (SW), 124 (PH), 93 (SY), 73 (MT), 65 (SN), 53 (NPB), 46 (PN), 28 (BY), 23 (PY), 21 (SL), 15 (TW), 13 (SPUA), 12 (FEBN), 10 (BH and LMI, each), 7 (SB, SID, and ST, each), 4 (PNMI and SV, each), 3 (SD) and 1 (BN, DTS, FBH and NRV, each). QTLs from different populations on the map displayed different positions. Several QTLs for the same traits were found across the map, for example, in *KenC-8 x N53-2* population, QTLs for SW were both present on A02 (2.15 and 22.11 Mb), on C07 (2.53 Mb) and on C08 (36.35 Mb). They could be located in separate regions or they could overlap with other populations’ QTLs. Thus, 92 regions that contained 198 overlapping QTLs were observed, they were located on 16 chromosomes and involved 11 traits and 17 populations which were developed in China, America and Germany (**Figure 1** and Supplementary Table 2).

**FIGURE 2:**
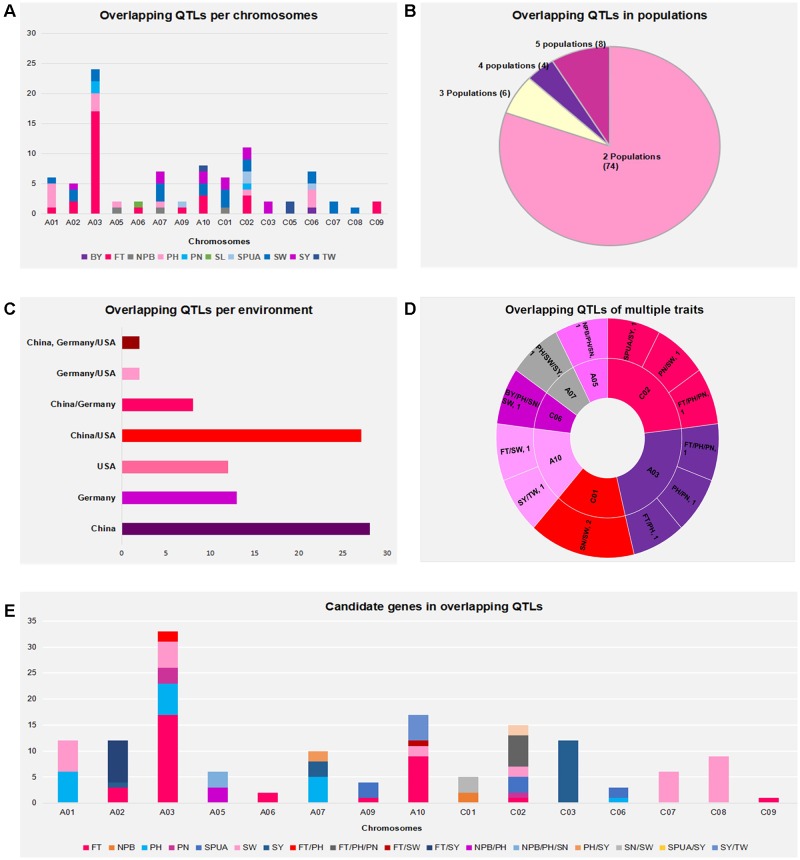
**(A)** Overlapping QTLs per chromosomes. Traits are labeled with different colors. **(B)** Overlapping QTLs in populations. **(C)** Overlapping QTLs per environment. **(D)** Overlapping QTLs of multiple traits. **(E)** Candidate genes detected in overlapping QTLs. Traits are labeled with different colors.

The present results revealed that QTLs from the Canadian population *Regent* × *Lagoda* did not overlap with any of the QTLs from other populations. In fact, while observing the genetic background of parental lines in these populations (known relatives are presented on **Table 1**), it is obvious that *Express617 × R53* and *Express617 × V8* shared a common parental line in *Express617*, as well as *MF216 × P1804, RV128 × P1804, RV289 × P1804*, and *TO1141 × P1804* which had *P1804* in common. Thus, overlapping QTLs from these populations were not surprising (e.g., overlapping SPUA QTLs *DH-slpa* from *Express617 × V8* and *Sil/dm2N16-2* from *Express617 × R53* on C6, overlapping SW QTLs *sw7.3* from *RV289 × P1804* and *sw7.1* from *RV128 × P1804* on A7). Further analysis revealed that regions of overlapping QTLs were mainly located on chromosome A03 (24/92), then on C02 (11/92), A10 and C01 (8/92, each), A7 and C06 (7/92, each), A01 (6/92), A02 (5/92), A05 (3/92), A06, A09, C03, C05, C07 and C09 (2/92, each), and C08 (1/92). The most frequently detected overlapping QTLs for each traits are presented on **Figure 2A**, among 92 regions of overlapping QTLs, 30 regions were for FT, 20 regions were for SW, 13 regions were for PH, 11 regions were for SY, 4 regions were for SPUA, 3 regions each were for NPB, PN, SN and TW, and 1 region each for BY and SL.

In addition, 74 of 92 regions of overlapping QTLs involved two populations (e.g., *RV289 × P1804* and *Tapidor × Ningyou7* for FT trait on A02), eight regions of overlapping QTLs involved five populations (e.g., *Tapidor × Ningyou7, MFDH × P124, RV128 × P1804, RVDH × P124* and 448 lines for FT trait on A03), six regions of overlapping QTLs involved three populations (e.g., *Express617 × V8, Express617 × R53* and *S1 × S2* for SW trait on A01), and four regions of overlapping QTLs involved four regions (e.g., *Tapidor × Ningyou7, RV128 × P1804, RVDH × P124* and 448 lines on A03) (**Figure 2B**). Moreover, 53 regions of overlapping QTLs were of environmental fixed QTLs: 28 among them were from the mapping population of Chinese environments, 13 and 12 among them were from German and American environments, respectively. The remaining 39 regions of overlapping QTLs were of mixed populations: QTLs from China and America overlapped the most (27/39 overlapping QTLs), and eight and two implied QTLs from China and Germany, and America and Germany, respectively, also since *“Sollux × Gaoyou”* was developed both in China and Germany, two overlapping QTLs implied the populations developed from America, China and Germany (**Figure 2C**). Finally, 13 regions of overlapping QTLs might affect multiple traits simultaneously: three regions were found on each of A03 (FT/PH, PH/PN, and FT/PH/PN) and C02 (FT/PH/PN, PN/SW, and SPUA/SY), two regions were found on each of A10 (FT/SW and SY/TW) and C01 (both SN/SW), and one region was found on each of A05 (NPB/PH/SN), A07 (PH/SW/SY) and C06 (BY/PH/SW) (**Figure 2D** and Supplementary Table 3). In overall, 57 regions of overlapping QTLs were located on A genome and 35 regions were on C genome. These results indicated that despite the difference in genetic × environment backgrounds, stable loci could be conserved in multiple populations of rapeseed. In addition, some loci might affect more than one beneficial trait, simultaneously.

### Regions of Overlapping QTLs Contained Potential Candidate Genes

A total of 1562 genes in *B. napus* were homologous of 425 yield related genes in *A. thaliana* (Supplementary Table 4). Then, 1398 among these genes were used to uncover potential candidate genes in overlapping QTLs, the remaining 164 genes could not be placed since their chromosome location were unknown. As result, a total of 147 candidate genes were found inside regions of overlapping QTLs for nine traits: FT, NPB, PH, PN, SN, SPUA, SW, SY, and TW (**Figure 2E** and Supplementary Table 5). Candidate genes for single trait were respectively of 34 (FT), 30 (SW), 18 (PH), 16 (SY), 8 (SPUA), 4 (PN), and 2 (NPB), and candidates for multiple traits were respectively of 8 (FT/SY), 6 (FT/PH/PN), 5 (SY/TW), 3 (NPB/PH, NPB/PH/SN, and SN/SW, each), 2 (PH/SY, SPUA/SY, and FT/PH, each), and 1 (FT/SW). Thus, 53 (FT), 34 (PH and SW, each), 33 (SY), 10 (PN and SPUA, each), 9 (SN), 8 (NPB), and 5 (TW) were discovered in this analysis. Further observation revealed that most of these candidate genes were on chromosome A03 (33/147), the other chromosomes contained 17 (A10), 15 (C02), 12 (A01, A02 and C03, each), 10 (A07), 9 (C08), 6 (A05 and C07, each), 5 (C01), 4 (A09), 3 (C06), 2 (A06) and 1 (C09) candidate genes, respectively. In this study, nine candidate genes were found inside region of overlapping QTLs which involved three traits: three genes on A05 (*BnaA05g07510D, BnaA05g07580D*, and *BnaA05g07740D*) that might affect NPB, PH and SN traits, six other genes on C2 (*BnaC02g01710D, BnaC02g45080D, BnaC02g45090D, BnaC02g02210D, BnaC02g45250D* and *BnaC02g02440D*) that possibly affect FT, PH and PN traits. Besides, candidate genes detected in *B. napus* often fell into QTL intervals of traits which were different from related function in *A. thaliana* orthologous genes. For example*, B. napus BnaC09g36060D* is homologous of *A. thaliana* RAX2 gene *AT2G36890* which was related to BN, however, *B. napus BnaC09g36060D* fell into overlapping QTLs of FT. These findings showed genes that might be responsible for one or multiple traits. Besides, homologous genes might affect dissimilar traits.

**FIGURE 3:**
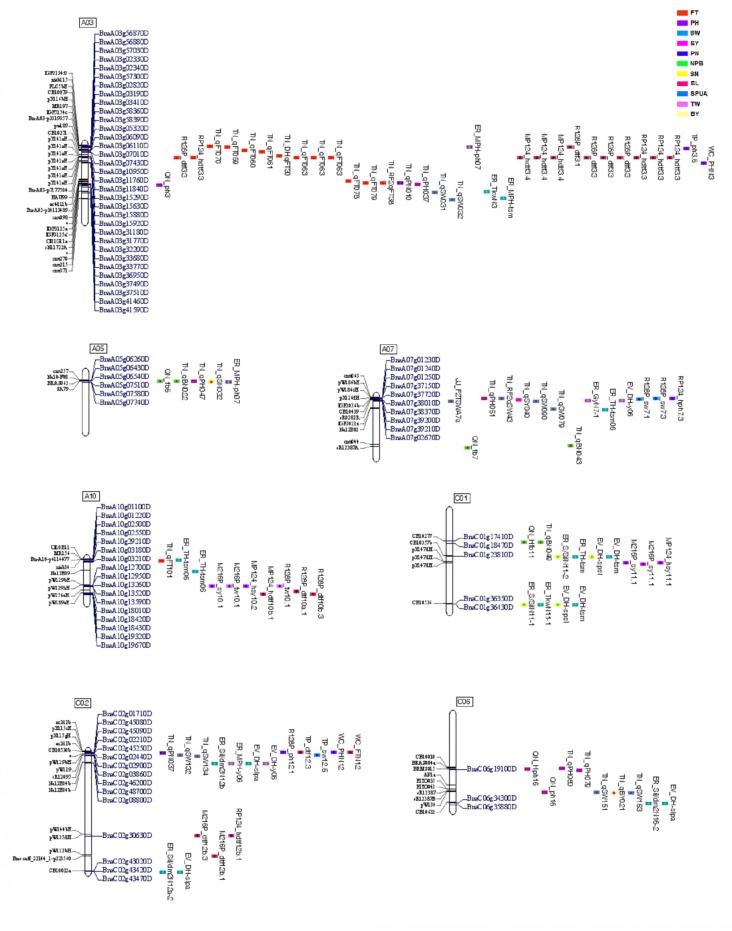
Regions of overlapping QTLs with multiple traits. These regions contained overlapping QTLs. Markers are on the left and QTL names and candidate genes are on the right. Different colors of QTL correspond to the 11 traits studied, and color of dots inside them correspond to the environment of origin (China in red, America in green, and Germany in blue). Code color of QTLs are on the right-side up of the figure.

Assumption from above-cited findings, genome of *B. napus* that might offer maximum benefit might correspond to a genome enriched with regions where QTLs of multiple traits overlapped, as seen on chromosomes A03, A05, A07, A10, C01, C02, and C06 where QTLs for two or three traits could overlap in the same regions, as mentioned previously (**Figure 3** and Supplementary Table 3).

### Candidate Genes Interaction Network Investigation Revealed Nine More Influential Genes of Diverse Metabolisms

**FIGURE 4:**
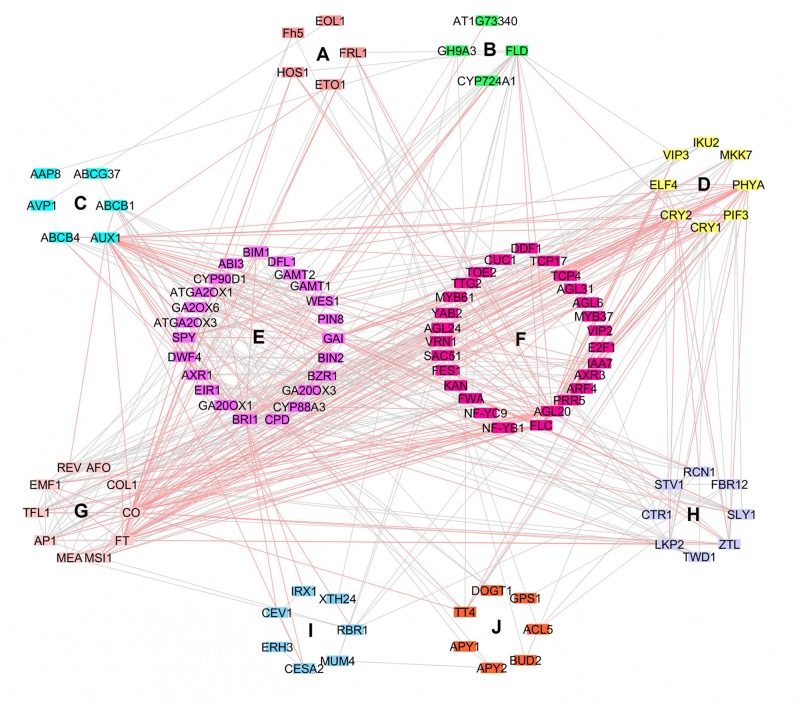
Interaction network of candidate genes related to yield traits. Genes are represented by nodes, nodes are connected by edges and clustered according to function in *A. thaliana*: **(A)** Cell, **(B)** Miscellaneous group, **(C)** Transport, **(D)** Signaling, **(E)** Hormone metabolism, **(F)** RNA regulation, **(G)** Development, **(H)** Protein metabolism, **(I)** Others: Stress, nucleotide metabolism, polyamine metabolism, secondary metabolism, **(J)** Unknown group. Nodes with their related pink edges indicate genes that interact the most with the other genes (DL≥20).

Interaction analysis between 147 candidate genes of *B. napus* were conducted using their 117 orthologous genes in *A. thaliana* (Supplementary Table 6). Gene ontology analysis indicated that these candidate genes could be divided into ten clusters, corresponding to 12 distinct functions which were clearly classified in the interaction network analysis (**Figure 4** and Supplementary Table 7): cell metabolism, transport, signaling, hormone metabolism, RNA regulation, development, protein metabolism, others (stress, nucleotide metabolism, polyamine metabolism, secondary metabolism), miscellaneous group, and an unknown group. The network contained 105 nodes and 449 edges. Obviously, nine genes that have dissimilar function might have higher influence over the other genes (degree layout, DL ≥ 20): AUX1 (transport, DL = 34), CO and FT (development, DL = 32 and 30, respectively), AGL20 and FLC (RNA regulation, DL = 26 and 24, respectively), CRY2 and PHYA (signaling, DL = 25 and 24, respectively), and BRI1 and GAI (hormone metabolism, DL = 24). In fact, functions of these nine influential candidate genes are related to flowering process, light response and plant hormones. In *B. napus*, these candidate genes fell into ten regions of overlapping QTLs, and might affect six traits, including SY, SW, PN, FT, PH, and NPB. These findings indicated that yield traits might be affected at multiple set of metabolisms.

## Discussion

### Unified Seed Yield and Yield-Related QTLs in One Map Revealed Important Stable Loci for Yield Improvement

In *B. napus*, SY depends on the number of PN or number of siliques per plant, the number of seeds per silique and the seed weight, these traits affect SY QTLs in a direct manner (Qzer et al., 1999; Quarrie et al., 2006; Chen et al., 2007). PN is a component in rapeseed that is targeted by breeders because it shows the highest correlation with yield, it is a sensitive quantitative trait, which shows extensive variation among cultivars and environment factors, thus it is important in genetic variation (Shi et al., 2015). SL and seed weight are both quantitative trait with high inheritance (Zhang and Zhou, 2006), and represent crucial keys for yield potentiality; thus, they are also targeted for breeding selection in rapeseed (Ali et al., 2003; Samizadeh et al., 2007). Furthermore, SY is indirectly influenced by traits, such as PH, first effective BH, first effective BN, LMI, PNMI, FT, MT, BY (Qiu et al., 2006; Shi et al., 2009; Zhou et al., 2014; Zhao et al., 2016). These traits are important to breeders because they show interaction with the SY (Zhang and Zhou, 2006); but also, they are environment sensitive traits, few stable QTLs were found in different environments in previous study (Shi et al., 2009).

In the present study, position of QTL in different populations were different, probably due to the genetic difference between parental lines, the type and size of the populations used, and the environments where they were developed (Zhu and Zhao, 2007). Overlapping QTLs emphasized regions on the genome which were responsible for trait variation and well conserved across the populations. Overlapping QTLs within *Express617 × R53* and *Express617 × V8* populations, *MF216 × P1804, RV128 × P1804, RV289 × P1804 and TO1141 × P1804* populations were not surprising, as they shared common ancestry which were *Express617* and *P1804*, respectively. Besides, *Regent* was among the 448 lines studied by Wang N. et al. (2016), it is surprising that no overlapping QTLs were found between QTLs detected in this study with the Canadian line *Regent × Lagoda*. Similarly, *RV128* and *Westar* were related, and no overlapping QTLs were found between *RV128 × P1804* and *Westar* (*Marnoo*)* × Ceres.* The remaining populations mentioned in this study had no common ancestry, in our knowledge. Otherwise, 53 regions of environment fixed QTLs could be deduced from the alignment map (e.g., 26/53 for China), despite the genotype difference of populations, similar QTL regions could be found due to the environment impact. Nevertheless, overlapping QTLs could be observed from populations of different environments (e.g., China/America), these regions might be enriched with related gene variations, but it is also possible that they share common ancestry. Besides, we could find QTLs which could not overlap with other QTLs (e.g., QTLs from Canadian populations *Regent × Lagoda*), they were specified QTLs which could not be on the same region as the others, probably due to the above-mentioned factors (difference in parents, environments, size of populations), but also the density of genetic map of which they were deduced. A region on A03 (from 4.25 to 6.05 Mb) had overlapping QTLs for FT, implying five populations, which were stable QTLs from populations of two different environments. Also, several regions of overlapping QTLs with multiple traits were found, these regions might be advantageous to develop for rapeseed breeding in targeting multiple traits simultaneously.

Besides, our analysis supported the importance of quantitative traits analysis in identification of SY and yield components. However, identification of the right gene combination in various loci is a challenging task, particularly when several loci with multiple alleles are associated. In this case, molecular plant breeding and genomics rather than conventional breeding will play a very significant role in increasing breeding efficiency. For example, sequencing of the *Brassica* genomes, candidate gene identification for controlling SY, and knowledge of the location of these specific genes in the genome and their roles will extend their importance in molecular-marker technology in plant breeding. This is because development of allele molecular markers from the gene itself can be synthesized. Findings of the current analysis are valuable to enhance breeding programs. Genes found within these QTLs intervals could be potential genes that might affect these traits and consequently could be manipulated to get the desired traits, notably producing high SY.

### Candidate Genes Revealed in the Region of Overlapping QTLs Could Be Used as Tool to Get Desired Traits

In the current study, a total of 147 candidate genes were found in 65 regions of overlapping QTLs, and several candidate genes might affect multiple traits at the same time. As mentioned above, three independent works were published on candidate genes for yield traits in *B. napus*: first, Zhao et al. (2016) found candidate genes for SY (*n* = 4), seed weight (*n* = 2), and PH (*n* = 1), by comparative mapping between *A. thaliana, B. rapa, B. oleracea* and *B. napus*, in 348 DH lines from cross between *KenC-8* and *N53-2*, they were grown in Dali, Shannxi for 5 years (Winter type) and Sunan, Gansu Province for 3 years (Spring type). Second, Zheng et al. (2017) discovered 31, 15, and 17 candidate genes for PH, branch initiation height and BN traits, respectively, by using of 333 diverse rapeseed accessions (20 are winter type, 308 are semi-winter type and 5 are unknown as referred to Sun et al., 2016), which were grown during 2012–2015 in Wuhan, China. They identified candidate genes by using gene sequences corresponding to *A. thaliana* and located on flanking regions of up to 450 kb on either side of significant SNPs markers, and then used them to GO annotations, with auxin, GA, IAA, SL, CK, and FT taken as the most likely candidate. Finally, Lu et al. (2017) identified candidate genes for SN (*n* = 6), PN per plant (*n* = 7), branch PN (*n* = 7), branch yield (*n* = 3), and thousand seed weight (*n* = 1), from a study led on 520 rapeseed accessions grown in Chongqing and Yunnan, by using GWAS and RNA-seq tools. Comparing our results with these cited papers, *BnaA02g02560D* was a candidate for SY in both our analysis and Zhao et al. (2016) analysis. Besides, three candidate genes for different traits: *BnaA02g02560D* was a candidate for SY in our analysis, however, it was a candidate for PH in Zheng et al. (2017) analysis, also *BnaA02g12130D* and *BnaA02g12260D* were candidates for FT in our analysis, and they were candidates for PH and branch initiation height in Zheng et al. (2017) analysis. However, none of candidate genes in Lu et al. (2017) analysis were similar to our findings. Since one gene might influence many traits and one trait might be influenced by many genes, as established in our analysis, hypotheses from our study and from these other studies might be both exact if proven by experimental approaches.

Identification of candidate genes in earlier time needed comparative mapping using *A. thaliana* as a model plant to map and discover genes in related species. For instance, Long et al. (2007) found *BnFLC010*, candidate gene underlying *qFT10-4* QTL, which was the key gene that controls differentiation of spring or winter rapeseed types based on these comparative mapping analyses. Furthermore, Ding et al. (2010) located the specific genes which controlled phosphorus concentration in the seeds, while the candidate genes controlling FT was discovered by Shi et al. (2011) using comparative mapping with the genome of *A. thaliana.* Advancement of these studies in order to ascertain the effects of the QTLs on different agronomic traits by use of comparative mapping technique have been undertaken by many research groups (Cai et al., 2014). Adoption of this technique is very significant in breeding for high yielding traits in *B. napus* cultivars. This is because it has made available abundant information that are useful in further understanding the genetic mechanisms of SY and yield related traits.

Nowadays, since the release of *B. napus* genome sequence, it is possible to identify candidate genes through *B. napus* physical map. Location of QTL flanking markers were searched on *B. napus* physical map via Blastn and E-PCR (Schuler, 1997; Rotmistrovsky et al., 2004), then the genes found inside the QTL regions are potential candidate genes. This technique has already been used to identify potential candidate genes in *B. napus*, for instance, Chao et al. (2017) used this technique to identify potential candidate genes for seed oil content trait, and found 448 genes underlying 41 oil content QTLs. Such kind of technique is also beneficial to identify candidate genes of QTLs from different linkage maps that could be could be combined in one map. Recently, we performed a meta-QTL analysis for fatty acid profile and oil content in *B. napus*, similar as this current study, we combined into one physical map many QTLs from different genetic and environmental background, then we identified 82 regions of overlapping QTLs and 218 candidate genes in 162 QTLs regions, 76/218 candidate genes were found inside 57 regions of overlapping QTLs, which were majorly for oil content traits (Raboanatahiry et al., 2017). Candidate genes of overlapping QTLs were supposed to be more stable rather than from independent QTL, different results might be obtained if candidate genes of independent QTLs were studied. Candidate genes are responsible for QTL and cause trait variation (Tabor et al., 2002). Thus, in our analysis, these candidate genes that might affect one or multiple traits could be used as tool to acquire any chosen traits in order to get high SY. They also can be used to get the most beneficial gene combination to get maximum profit, especially those genes which were found in region of overlapping QTLs involving many traits.

### Candidate Genes Interaction Network Indicated That Yield Might Be Affected at Different Set of Metabolisms

In our analysis, QTLs from different genotype × environment background had different location on the physical map of *Darmor-bzh*, and this affect also the candidate genes discovered during this study. Thus, the study of genetic network allows to comprehend the interaction between genes and the mechanism that works for the biological system. Besides, quantitative traits are influenced by dynamism and structure of genetic network (Frank, 2003). In this study, candidate genes interaction network was made from homologous genes in *A. thaliana*, since *B. napus* is not accessible on STRING Database. Actually, *B. rapa* (A genome) is available on STRING database but is not enough to provide more information for this study compared to *A. thaliana*. First, homologous genes in *B. rapa* would represent only a part of detected candidate genes in *B. napus* and this would distort the results. Second, annotated and cloned genes were rare in *B. rapa* than in *A. thaliana*, thus taking *A. thaliana* as reference would help greatly in comprehension of the interaction of candidate genes based on their functions. This strategy was used previously to investigate on proteins interaction in other studies (Gu et al., 2016; Chao et al., 2017; Raboanatahiry et al., 2017).

Thus, nine genes (AUX1, CO, FT, FLC, PHYA, CRY2, AGL20, BRI1, GAI) which are involved in important functions (flowering process, light response, and plant hormones) might have higher influence on the other genes which possibly dependent on these nine genes. CO, FT, FLC and AGL20 were four key genes in the network interaction of our analysis and they are both implicated in plant flowering development. CONSTANS (CO) encodes a transcription factor that induces flowering in long exposure to daylight and stimulates the expression of FLOWERING LOCUS (FT) in phloem of leaves (Koornneef et al., 1991; Putterill et al., 1995; Suárez-López et al., 2001; An et al., 2004; Smit and Weijers, 2015). It has been shown recently that CO interacted with two VASCULAR PLANT ONE-ZINC FINGER (VOZ1 and VOZ2) and indorsed transition of photoperiodic flowering (Kumar et al., 2018). Note that FT is induced in long daylength and promotes flowering (Li C. et al., 2011). In fact, FT is the major component of florigen and promotes the transmission of flowering signal from leaf to shoot, where FT forms a complex with FD (bZIP transcription factor) and activate meristem genes to promote flowering (Abe et al., 2005; Wigge et al., 2005; Corbesier et al., 2007; Zeevaart, 2008; Nakamura et al., 2014). However, FLOWERING LOCUS C (FLC), which is a dominant transcription factor gene implied in vernalization, is antagonistic to FT (Michaels and Amasino, 1999; Sheldon et al., 1999; Hepworth et al., 2002; Helliwell et al., 2006). In fact, FLC blocks expression of both FT, FD and SUPPRESSOR OF CONSTANS 1 (SOC1) in meristem, which consequently abort flower genesis (Lee et al., 2000; Onouchi et al., 2000; Samach et al., 2000; Searle et al., 2006). Nevertheless, a gene that encodes a MADS-domain protein, named AGAMOUS-LIKE 20 (AGL20) is positively regulated by unsolicited vernalization, and independent flowering and photoperiodic pathways, and could relieve late flowering in plants that hold FLC functional alleles (Lee et al., 2000). PHYTOCHROME A (PHYA) and CRYPTOCHROME2 (CRY2) were also key genes in interaction network of our analysis, they could also encourage flowering by CO stabilization via CONSTITUTIVE PHOTOMORPHOGENIC 1 (COP1) and SUPPRESSOR OF PHYA1 (SPA1) ubiquitin ligase complex in nightfall (Sarid-Krebs et al., 2015). Light-labile PHYA is the most abundant phytochrome in etiolated seedlings and could absorb red, far-red light and blue/ultraviolet-A wavelengths (Sharrock and Quail, 1989; Li J. et al., 2011). PHYA could interact with SPA and TANDEM ZINC-FINGER/PLUS3 (TZP), disrupting COP1/SPA complex to induce photomorphogenesis, and regulating PHYA abundance and phosphorylation in far red light, respectively (Sheerin et al., 2015; Zhang S. et al., 2018). However, CRY2 are blue light receptors and intercede in response to photoperiodic flowering, leaf senescence and cell development, hypocotyl elongation and flowering regulations (Ahmad and Cashmore, 1993; Guo et al., 1998; Lin et al., 1998; El-Assal et al., 2001; Cashmore, 2003; Meng et al., 2013). AUX1, BRI1 and GAI are genes related to plant hormones which were detected as highly influential in our interaction network analysis. AUXIN INFLUX TRANSPORTER1/AUXIN RESISTANT1 (AUX1) brings auxin hormone to the root apex and intercedes in repression of root elongation in presence of heavy metals and alkalinity, and in control of cytokinin on root gravitropism (Swarup et al., 2001; Sun et al., 2010; Li et al., 2015; Pernisova et al., 2016; Yuan and Huang, 2016). Besides, BRASSINOSTEROID INSENSITIVE1 (BRI1) recognizes brassinosteroids hormone (BRs) in growth and development regulation, and endocytosis of BRI1 is important to reduce signaling; if incorporation of BRI1 is inhibited, BRs response is enhanced, and in case of ubiquitination loss, degradation of BRI1 is hindered (Li and Chory, 1997; Irani et al., 2012; Zhou et al., 2018). Also, GIBBERELLIC ACID INSENSITIVE (GAI), which is a DELLA motif containing protein, is involved in regulation of plant growth and in inhibition of GA-mediated plant responses (Yamaguchi, 2008; Davière and Achard, 2013). With REPRESSOR OF GA1-3 (RGA), GAI might have overlapping roles in senescence regulation by weakening transcription factor WRKY6 activity on senescence related genes (Zhang Y. et al., 2018).

Actually, appropriate FT greatly influences the potential of rapeseed yield (Luo et al., 2014), it could have impact on hybrid vigor, and consequently on reproductive success (Srikanth and Schmid, 2011). FT is significantly related to maturity (Miguel et al., 1998; Searle et al., 2006), and the optimal time depends on the cultivar (i.e., winter or spring type) and the environment of cultivation. The identification of genes which control FT might offer sustainable agriculture according to environmental variation. Multiple genetic pathways that respond to different development and environment conditions control the floral transition (Baurle and Dean, 2006; Srikanth and Schmid, 2011; Andres and Coupland, 2012). In *A. thaliana*, five main pathways were found to control flowering: the gibberellin pathway in which flowering needs the activity of gibberellic acid; the endogenous pathway which modulates flowering according to the age of plant; the photoperiod and the vernalization pathways which control flowering according to day length and temperature fluctuation, respectively; and the autonomous pathway which works independently of gibberellin and photoperiod pathways (Amasino and Michaels, 2010; Srikanth and Schmid, 2011). The keys genes of our interaction network were involved in these pathways. Note that in a study which aimed to evaluate rapeseed for yield performance, it has been demonstrated that early flowering promoted better SY because it gave enough time for the seeds to fill (Ali et al., 2013).

Otherwise, these nine above-mentioned genes might affect variation of traits, and quantitative traits are affected by structure and dynamism of genetic regulatory networks (Tabor et al., 2002; Frank, 2003). In the current interaction network analysis, candidate genes from overlapping QTLs of multiple population were taken to the study to find the nine above-mentioned pivotal genes. Other findings might be found if investigation was made on single population. At the end, these findings indicated that yield traits might be affected at multiple set of metabolisms. Unfortunately, in our analysis, we used *A. thaliana* but not *B. napus*, which indicated that findings might be imprecise. Nevertheless, construction of this network gave new insight on interaction of candidate genes.

## Conclusion

This study reported QTLs for yield and yield-related traits in *B. napus*, which could be aligned and combined in one physical map. Unluckily, some QTLs could not be aligned in the map. However, stable QTLs which corresponded to overlapping QTLs could be predicted, but need confirmation. Nevertheless, region enriched with QTL were found which could help to understand the process of close linkage in traits and to identify varied or conserved area. Then, this map could serve as reference to understand rapeseed genome characteristic, especially while studying yield traits. Additionally, new candidate genes could be detected in these hypothetical stable QTLs, but still need to be confirmed as well, even if they were carefully chosen due to their presupposed yield related function and their position within QTL regions. Based on our current study, markers could be developed and potential candidate genes could be tested for breeding improvement purpose.

## Author Contributions

NR and HC wrote the manuscript. NR, HC, HD, SP, and WY performed the analysis of QTL alignment map. NR analyzed the candidate genes and interaction network. ML, BW, and LY supervised the work, and revised the manuscript.

## Conflict of Interest Statement

The authors declare that the research was conducted in the absence of any commercial or financial relationships that could be construed as a potential conflict of interest.

## Footnotes

1 http://www.brassica.info/resource/maps/lg-assignments.php

2 http://www.ncbi.nlm.nih.gov/

3 http://brassica.nbi.ac.uk/IGF/

4 http://archive.gramene.org/markers/

5 http://string-db.org/

## Abbreviations

BH: branch height
BN: branch number
BY: biomass yield
DTS: development time of seeds
FBH: first branch height
FEBN: first effective branch number
FT: flowering time/days to flowering
LMI: length of main inflorescence
MT: maturity time
NPB: number of primary branches
NRV: Napus root vigor
PH: plant height
PN: pod number/silique per plant
PNMI: pod number of main inflorescence
PY: pod yield/100pods
SB: silique breadth
SD: seed density
SID: silique density
SL: silique length
SN: seed number per pod/Seeds per silique
SPUA: Silique per unit area
ST: silique thickness
SV: silique volume
SW: seed weight/thousand seed weight
SY: seed yield
TW: test weight

## References

[B1] Abe M. Kobayashi Y. Yamamoto S. Daimon Y. Yamaguchi A. Ikeda Y. (2005). FD, a bZIP protein mediating signals from the floral pathway integrator FT at the shoot apex. *Science* 309 1052–1056. 10.1126/science.1115983 16099979

[B2] Ahmad M. Cashmore A. R. (1993). HY4 gene of *A. thaliana* encodes a protein with characteristics of a blue-light photoreceptor. *Nature* 366 162–166. 10.1038/366162a0 8232555

[B3] Ali I. Ahmed H. M. Shah S. A. (2013). Evaluation and selection of rapeseed (*Brassica napus* L.) mutant lines for yield performance using augmented design. *J. Anim. Plant Sci.* 23 1125–1130.

[B4] Ali N. Javidfar F. Elmira J. Y. Mirza M. Y. (2003). Relationship among yield components and selection criteria for yield improvement in winter rapeseed (*Brassica napus* L.). *Pak. J. Bot.* 35 167–174. 10.1016/S1671-2927(11)60086-2

[B5] Al-Shehbaz P. I. A. Beilstein M. A. Kellogg E. (2006). Systematics and phylogeny of the Brassicaceae (Cruciferae): an overview. *Plant Syst. Evol.* 259 89–120. 10.1007/s00606-006-0415-z

[B6] Amasino R. M. Michaels S. D. (2010). The timing of flowering. *Plant Physiol.* 154 516–520. 10.1104/pp.110.161653 20921176 PMC2948982

[B7] An H. Roussot C. Suárez-López P. Corbesier L. Vincent C. Piñeiro M. (2004). CONSTANS acts in the phloem to regulate a systemic signal that induces photoperiodic flowering of *Arabidopsis*. *Development* 131 3615–3626. 10.1242/dev.01231 15229176

[B8] Andres F. Coupland G. (2012). The genetic basis of ?owering responses to seasonal cues. *Nat. Rev. Genet.* 13 627–639. 10.3410/f.717963417.793465299 22898651

[B9] ArifUzZaman M. Mamidi S. McClean P. Rahman M. (2016). QTL mapping for root vigor and days to flowering in *Brassica napus* L. *Can. J. Plant Sci.* 97 99–109. 10.1139/cjps-2016-0048

[B10] Bailey C. D. Koch M. A. Mayer M. Mummenhoff K. O’kane S. L. Warwick S. I. (2006). Toward a global phylogeny of the Brassicaceae. *Mol. Biol. Evol.* 23 2142–2160. 10.1093/molbev/msl087 16916944

[B11] Basunanda P. Radoev M. Ecke W. Friedt W. Becker H. C. Snowdon R. J. (2010). Comparative mapping of quantitative trait loci involved in heterosis for seedling and yield traits in oilseed rape (*Brassica napus* L.). *Theor. Appl. Genet.* 120 271–281. 10.1007/s00122-009-1133-z 19707740 PMC2793389

[B12] Baurle I. Dean C. (2006). The timing of developmental transitions in plants. *Cell* 125 655–664. 10.1016/j.cell.2006.05.005 16713560

[B13] Butruille D. V. Guries R. P. Osborn T. C. (1999). Linkage analysis of molecular markers and quantitative trait loci in populations of inbred backcross lines of *Brassica napus* L. *Genetics* 153 949–964. 10511570 10.1093/genetics/153.2.949 PMC1460775

[B14] Cai D. Xiao Y. Yang W. Ye W. Wang B. Younas M. (2014). Association mapping of six yield-related traits in rapeseed (*Brassica napus* L.). *Theor. Appl. Genet.* 127 85–96. 10.1007/s00122-013-2203-9 24121524

[B15] Cashmore A. R. (2003). Cryptochromes: enabling plants and animals to determine circadian time. *Cell* 114 537–543. 10.1016/j.cell.2003.08.004 13678578

[B16] Chalhoub B. Denoeud F. Liu S. Parkin I. A. Tang H. Wang X. (2014). Early allopolyploid evolution in the post-Neolithic *Brassica napus* oilseed genome. *Science* 345 950–953. 10.1126/science.1253435 25146293

[B17] Chao H. Wang H. Wang X. Guo L. Gu J. Zhao W. (2017). Genetic dissection of seed oil and protein content and identification of network associated with oil content in *Brassica napus*. *Sci. Rep.* 7:46295. 10.1038/srep46295 28393910 PMC5385559

[B18] Chen W. Zhang Y. Liu X. Chen B. Tu J. Fu T. (2007). Detection of QTL for six yield-related traits in oilseed rape (*Brassica napus*) using DH and immortalized F2 populations. *Theor. Appl. Genet.* 115 849–858. 10.1007/s00122-007-0613-2 17665168

[B19] Corbesier L. Vincent C. Jang S. Fornara F. Fan Q. Searle I. (2007). FT protein movement contributes to long-distance signaling in floral induction of *Arabidopsis*. *Science* 316 1030–1033. 10.1126/science.1141752 17446353

[B20] Davière J. M. Achard P. (2013). Gibberellin signaling in plants. *Development* 140 1147–1151. 10.1242/dev.087650 23444347

[B21] Ding G. Yang M. Hu Y. Liao Y. Shi L. Xu F. (2010). Quantitative trait loci affecting seed mineral concentrations in *Brassica napus* grown with contrasting phosphorus supplies. *Ann. Bot.* 105 1221–1234. 10.1093/aob/mcq050 20237116 PMC2887070

[B22] El-Assal S. E. D. Alonso-Blanco C. Peeters A. J. Raz V. Koornneef M. (2001). A QTL for flowering time in *Arabidopsis* reveals a novel allele of CRY2. *Nat. Genet.* 29 435–440. 10.1038/ng767 11726930

[B23] Engqvist G. M. Becker H. C. (1991). Heterosis and epistasis in rapeseed estimated from generation means. *Euphytica* 58 31–35. 10.1007/BF00035337

[B24] Fan C. Cai G. Qin J. Li Q. Yang M. Wu J. (2010). Mapping of quantitative trait loci and development of allele-specific markers for seed weight in *Brassica napus*. *Theor. Appl. Genet.* 121 1289–1301. 10.1007/s00122-010-1388-4 20574694

[B25] Frank S. A. (2003). Genetic variation of polygenic characters and the evolution of genetic degeneracy. *J. Evol. Biol.* 16 138–142. 10.1046/j.1420-9101.2003.00485.x 14635888

[B26] Fu Y. Wei D. Dong H. He Y. Cui Y. Mei J. (2015). Comparative quantitative trait loci for silique length and seed weight in *Brassica napus*. *Sci. Rep.* 5:14407. 10.1038/srep14407 26394547 PMC4585775

[B27] Gu J. Chao H. Gan L. Guo L. Zhang K. Li Y. (2016). Proteomic dissection of seed germination and seedling establishment in *Brassica napus*. *Front. Plant Sci.* 7:1482. 10.3389/fpls.2016.01482 27822216 PMC5075573

[B28] Guo H. Yang H. Mockler T. C. Lin C. (1998). Regulation of flowering time by *Arabidopsis* photoreceptors. *Science* 279 1360–1363. 10.1126/science.279.5355.1360 9478898

[B29] Helliwell C. A. Wood C. C. Robertson M. Peacock W. J. Dennis E. S. (2006). The *Arabidopsis* FLC protein interacts directly in vivo with SOC1 and FT chromatin and is part of a high-molecular weight protein complex. *Plant J.* 46 183–192. 10.1111/j.1365-313X.2006.02686.x 16623882

[B30] Hepworth S. R. Valverde F. Ravenscroft D. Mouradov A. Coupland G. (2002). Antagonistic regulation of flowering-time gene SOC1 by CONSTANS and FLC via separate promoter motifs. *EMBO J.* 21 4327–4337. 10.1093/emboj/cdf432 12169635 PMC126170

[B31] Irani N. G. Di Rubbo S. Mylle E. Van den Begin J. Schneider-Pizoń J. Hniliková J. (2012). Fluorescent castasterone reveals BRI1 signaling from the plasma membrane. *Nat. Chem. Biol.* 8 583–589. 10.1038/nchembio.958 22561410

[B32] Jesske T. Olberg B. Schierholt A. Becker H. C. (2013). Resynthesized lines from domesticated and wild *Brassica* taxa and their hybrids with *B. napus* L.: genetic diversity and hybrid yield. *Theor. Appl. Genet.* 126 1053–1065. 10.1007/s00122-012-2036-y 23328861 PMC3607727

[B33] Koornneef M. Hanhart C. J. van der Veen J. H. (1991). A genetic and physiological analysis of late flowering mutants in *Arabidopsis* thaliana. *Mol. Genet. Genomics* 229 57–66. 10.1007/BF00264213 1896021

[B34] Kramer C. C. Polewicz H. Osborn T. C. (2009). Evaluation of QTL alleles from exotic sources for hybrid seed yield in the original and different genetic backgrounds of spring-type *Brassica napus* L. *Mol. Breed.* 24 419–431. 10.1007/s11032-009-9303-x

[B35] Krzywinski M. Schein J. Birol I. Connors J. Gascoyne R. Horsman D. (2009). Circos: an information aesthetic for comparative genomics. *Genome Res. Adv.* 19 1639–1645. 10.1101/gr.092759.109 19541911 PMC2752132

[B36] Kumar S. Choudhary P. Gupta M. Nath U. (2018). Vascular plant one-zinc finger1 (VOZ1) and VOZ2 interact with CONSTANS and promote photoperiodic flowering transition. *Plant Physiol.* 176 2917–2930. 10.1104/pp.17.01562 29507119 PMC5884617

[B37] Lee H. Suh S. S. Park E. Cho E. Ahn J. H. Kim S. G. (2000). The AGAMOUS-LIKE 20 MADS domain protein integrates floral inductive pathways in *Arabidopsis*. *Genes Dev.* 14 2366–2376. 10.1101/gad.813600 10995392 PMC316936

[B38] Li C. Gu M. Shi N. Zhang H. Yang X. Osman T. (2011). Mobile FT mRNA contributes to the systemic florigen signalling in floral induction. *Sci. Rep.* 1:73. 10.1038/srep00073 22355592 PMC3216560

[B39] Li J. Chory J. (1997). A putative leucine-rich repeat receptor kinase involved in brassinosteroid signal transduction. *Cell* 90 929–938. 10.1016/S0092-8674(00)80357-8 9298904

[B40] Li J. Li G. Wang H. Deng X. W. (2011). Phytochrome signaling mechanisms. *Arabidopsis Book* 9:e0148. 10.1199/tab.0148 22303272 PMC3268501

[B41] Li J. Xu H. H. Liu W. C. Zhang X. W. Lu Y. T. (2015). Ethylene inhibits root elongation during alkaline stress through AUXIN1 and associated changes in auxin accumulation. *Plant Physiol.* 168 1777–1791. 10.1104/pp.15.00523 26109425 PMC4528753

[B42] Lin C. Yang H. Guo H. Mockler T. Chen J. Cashmore A. R. (1998). Enhancement of blue-light sensitivity of *Arabidopsis* seedlings by a blue light receptor cryptochrome 2. *Proc. Natl. Acad. Sci. U.S.A.* 95 2686–2690. 10.1073/pnas.95.5.2686 9482948 PMC19462

[B43] Liu S. Fan C. Li J. Cai G. Yang Q. Wu J. (2016). A genome wide association study reveals novel elite allelic variations in seed oil content of *Brassica napus*. *Theor. Appl. Genet.* 129 1203–1215. 10.1007/s00122-016-2697-z 26912143

[B44] Long Y. Shi J. Qiu D. Li R. Zhang C. Wang J. (2007). Flowering time quantitative trait loci analysis of oilseed *Brassica* in multiple environments and genome-wide alignment with *Arabidopsis*. *Genetics* 177 2433–2444. 10.1534/genetics.107.080705 18073439 PMC2219480

[B45] Lu K. Peng L. Zhang C. Lu J. Yang B. Xiao Z. (2017). Genome-wide association and transcriptome analyses reveal candidate genes underlying yield-determining traits in *Brassica napus*. *Front. Plant Sci.* 8:206. 10.3389/fpls.2017.00206 28261256 PMC5309214

[B46] Luo Y. X. Luo C. Y. Du D. Z. Fu Z. Yao Y. M. Xu C. C. (2014). Quantitative trait analysis of flowering time in spring rapeseed (B. *napus L.)*. *Euphytica* 200 321–335. 10.1007/s10681-014-1140-2

[B47] Luo Z. Wang M. Long Y. Huang Y. Shi L. Zhang C. (2017). Incorporating pleiotropic quantitative trait loci in dissection of complex traits: seed yield in rapeseed as an example. *Theor. Appl. Genet.* 130 1569–1585. 10.1007/s00122-017-2911-7 28455767 PMC5719798

[B48] Meng Y. Li H. Wang Q. Liu B. Lin C. (2013). Blue light-dependent interaction between cryptochrome 2 and CIB1 regulates transcription and leaf senescence in soybean. *Plant Cell* 25 4405–4420. 10.1105/tpc.113.116590 24272488 PMC3875726

[B49] Michaels S. D. Amasino R. M. (1999). FLOWERING LOCUS C encodes a novel MADS domain protein that acts as a repressor of flowering. *Plant Cell* 11 949–956. 10.1105/tpc.11.5.949 10330478 PMC144226

[B50] Miguel A. Green R. Nilsson O. Michael R. Weigel D. (1998). Gibberellins promote flowering of *Arabidopsis* by activating the LEAFY promoter. *Plant Cell* 10 791–800. 10.1105/tpc.10.5.791 9596637 PMC144373

[B51] Nagaharu U. (1935). Genome analysis in *Brassica* with special reference to the experimental formation of *B. napus* and peculiar mode of fertilization. *Jpn. J. Bot.* 7 389–452.

[B52] Nakamura Y. Andrés F. Kanehara K. Liu Y. C. Dörmann P. Coupland G. (2014). *Arabidopsis* florigen FT binds to diurnally oscillating phospholipids that accelerate flowering. *Nat. Commun.* 5:3553. 10.1038/ncomms4553 24698997 PMC3988816

[B53] Nesi N. Delourme R. Bregeon M. Falentin C. Renard M. (2008). Genetic and molecular approaches to improve nutritional value of *Brassica napus* L. seed. *C. R. Biol.* 331 763–771. 10.1016/j.crvi.2008.07.018 18926490

[B54] Onouchi H. Igeno M. I. Perilleux C. Graves K. Coupland G. (2000). Mutagenesis of plants overexpressing CONSTANS demonstrates novel interactions among *Arabidopsis* flowering-time genes. *Plant Cell* 12 885–900. 10.1105/tpc.12.6.885 10852935 PMC149091

[B55] Paran I. Zamir D. (2003). Quantitative traits in plants: beyond the QTL. *Trends Genet.* 19 303–306. 10.1016/S0168-9525(03)00117-3 12801720

[B56] Pernisova M. Prat T. Grones P. Harustiakova D. Matonohova M. Spichal L. (2016). Cytokinins influence root gravitropism via differential regulation of auxin transporter expression and localization in *Arabidopsis*. *New Phytol.* 212 497–509. 10.1111/nph.14049 27322763

[B57] Putterill J. Robson F. Lee K. Simon R. Coupland G. (1995). The CONSTANS gene of *Arabidopsis* promotes flowering and encodes a protein showing similarities to zinc finger transcription factors. *Cell* 80 847–857. 10.1016/0092-8674(95)90288-0 7697715

[B58] Qi L. Mao L. Sun C. Pu Y. Fu T. Ma C. (2014). Interpreting the genetic basis of silique traits in *Brassica napus* using a joint QTL network. *Plant Breed.* 133 52–60. 10.1111/pbr.12131

[B59] Qiu D. Morgan C. Shi J. Long Y. Liu J. Li R. (2006). A comparative linkage map of oilseed rape and its use for QTL analysis of seed oil and erucic acid content. *Theor. Appl. Genet.* 114 67–80. 10.1007/s00122-006-0411-2 17033785

[B60] Quarrie S. A. Pekic Quarrie S. Radosevic R. Rancic D. Kaminska A. Barnes J. D. (2006). Dissecting a wheat QTL for yield present in a range of environments: from the QTL to candidate genes. *J. Exp. Bot.* 57 2627–2637. 10.1093/jxb/erl026 16831847

[B61] Quijada P. A. Udall J. A. Lambert B. Osborn T. C. (2006). Quantitative trait analysis of seed yield and other complex traits in hybrid spring rapeseed (*Brassica napus* L.): 1. Identification of genomic regions from winter germplasm. *Theor. Appl. Genet.* 113 549–561. 10.1007/s00122-006-0323-1 16767447

[B62] Qzer H. Oral E. Dogru U. (1999). Relationships between yield and yield components on currently improved spring rapeseed cultivars. *Turk. J. Agric. For.* 53 603–607.

[B63] Raboanatahiry N. Chao H. B. Guo L. Gan J. Xiang J. Yan M. (2017). Synteny analysis of genes and distribution of loci controlling oil content and fatty acid profile based on QTL alignment map in *Brassica napus*. *BMC Genomics* 18:776. 10.1186/s12864-017-4176-6 29025408 PMC5639739

[B64] Radoev M. Becker H. C. Ecke W. (2008). Genetic analysis of heterosis for yield and yield components in rapeseed (*Brassica napus* L.) by quantitative trait locus mapping. *Genetics* 179 1547–1558. 10.1534/genetics.108.089680 18562665 PMC2475754

[B65] Raman H. Raman R. Kilian A. Detering F. Long Y. Edwards D. (2013). A consensus map of rapeseed (*Brassica napus* L.) based on diversity array technology markers: applications in genetic dissection of qualitative and quantitative traits. *BMC Genomics* 14:277. 10.1186/1471-2164-14-277 23617817 PMC3641989

[B66] Remington D. L. Purugganan M. D. (2003). Candidate gene, quantitative loci, and functional trait evolution in plants. *Int. J. Plant Sci.* 164 S7–S20. 10.1086/367812

[B67] Rotmistrovsky K. Jang W. Schuler G. D. (2004). A web server for performing electronic PCR. *Nucleic Acids Res.* 32 W108–W112. 10.1093/nar/gkh450 15215361 PMC441588

[B68] Samach A. Onouchi H. Gold S. E. Ditta G. S. Schwarz-Sommer Z. Yanofsky M. F. (2000). Distinct roles of CONSTANS target genes in reproductive development of *Arabidopsis*. *Science* 288 1613–1616. 10.1126/science.288.5471.1613 10834834

[B69] Samizadeh H. Samadi B. Y. Behamta M. R. Taleii A. Stringam G. R. (2007). Study of pod length trait in doubled haploid *Brassica napus* population by molecular markers. *J. Agric. Sci. Technol.* 9 129–136.

[B70] Sarid-Krebs L. Panigrahi K. C. Fornara F. Takahashi Y. Hayama R. Jang S. (2015). Phosphorylation of CONSTANS and its COP1-dependent degradation during photoperiodic flowering of *Arabidopsis*. *Plant J.* 84 451–463. 10.1111/tpj.13022 26358558

[B71] Schuler G. D. (1997). Sequence mapping by electronic PCR. *Genome Res.* 7 541–550. 10.1101/gr.7.5.541 9149949 PMC310656

[B72] Searle I. He Y. Turck F. Vincent C. Fornara F. Krober S. (2006). The transcription factor FLC confers a flowering response to vernalization by repressing meristem competence and systemic signaling in *Arabidopsis*. *Genes Dev.* 20 898–912. 10.1101/gad.373506 16600915 PMC1472290

[B73] Shannon P. Markiel A. Ozier O. Baliga N. S. Wang J. T. Ramage D. (2003). Cytoscape: a software environment for integrated models of biomolecular interaction networks. *Genome Res.* 13 2498–2504. 10.1101/gr.1239303 14597658 PMC403769

[B74] Sharrock R. A. Quail P. H. (1989). Novel phytochrome sequences in *Arabidopsis thaliana*: structure, evolution, and differential expression of a plant regulatory photoreceptor family. *Genes Dev.* 3 1745–1757. 10.1101/gad.3.11.1745 2606345

[B75] Sheerin D. J. Menon C. zur Oven-Krockhaus S. Enderle B. Zhu L. Johnen P. (2015). Light-activated phytochrome A and B interact with members of the SPA family to promote photomorphogenesis in *Arabidopsis* by reorganizing the COP1/SPA complex. *Plant Cell* 27 189–201. 10.1105/tpc.114.134775 25627066 PMC4330587

[B76] Sheldon C. C. Burn J. E. Perez P. P. Metzger J. Edwards J. A. Peacock W. J. (1999). The FLF MADS box gene: a repressor of flowering in *Arabidopsis* regulated by vernalization and methylation. *Plant Cell* 11 445–458. 10.2307/3870872 10072403 PMC144185

[B77] Shi J. Li R. Qiu D. Jiang C. Long Y. Morgan C. (2009). Unraveling the complex trait of crop yield with quantitative trait loci mapping in *Brassica napus*. *Genetics* 182 851–861. 10.1534/genetics.109.101642 19414564 PMC2710164

[B78] Shi J. Zhan J. Yang Y. Ye J. Huang S. Li R. (2015). Linkage and regional association analysis reveal two new tightly-linked major-QTLs for pod number and seed number per pod in rapeseed (*Brassica napus* L.). *Sci. Rep.* 5:14481. 10.1038/srep14481 26434411 PMC4593047

[B79] Shi Y. Zhang X. Xu Z. Y. Li L. Zhang C. Schläppi M. (2011). Influence of EARLI1-like genes on flowering time and lignin synthesis of *Arabidopsis thaliana*. *Plant Biol.* 13 731–739. 10.1111/j.1438-8677.2010.00428.x 21815977

[B80] Smit M. E. Weijers D. (2015). The role of auxin signaling in early embryo pattern formation. *Curr. Opin. Plant Biol.* 28 99–105. 10.1016/j.pbi.2015.10.001 26495766

[B81] Srikanth A. Schmid M. (2011). Regulation of flowering time: all roads lead to Rome. *Cell. Mol. Life Sci.* 68 2013–2037. 10.1007/s00018-011-0673-y 21611891 PMC11115107

[B82] Suárez-López P. Wheatley K. Robson F. Onouchi H. Valverde F. Coupland G. (2001). CONSTANS mediates between the circadian clock and the control of flowering in *Arabidopsis*. *Nature* 410 1116–1120. 10.1038/35074138 11323677

[B83] Sun F. Liu J. Hua W. Sun X. Wang X. Wang H. (2016). Identification of stable QTLs for seed oil content by combined linkage and association mapping in *Brassica napus*. *Plant Sci.* 252 388–399. 10.1016/j.plantsci.2016.09.001 27717475

[B84] Sun P. Tian Q. Y. Chen J. Zhang W. H. (2010). Aluminium-induced inhibition of root elongation in *Arabidopsis* is mediated by ethylene and auxin. *J. Exp. Bot.* 61 347–356. 10.1093/jxb/erp306 19858117 PMC2803203

[B85] Swarup R. Friml J. Marchant A. Ljung K. Sandberg G. Palme K. (2001). Localization of the auxin permease AUX1 suggests two functionally distinct hormone transport pathways operate in the *Arabidopsis* root apex. *Genes Dev.* 15 2648–2653. 10.1101/gad.210501 11641271 PMC312818

[B86] Szklarczyk D. Franceschini A. Wyder S. Forslund K. Heller D. Huerta-Cepas J. (2015). STRING v10: protein-protein interaction networks, integrated over the tree of life. *Nucleic Acids Res.* 43 D447–D452. 10.1093/nar/gku1003 25352553 PMC4383874

[B87] Tabor H. K. Risch N. J. Myers R. M. (2002). Candidate-gene approaches for studying complex genetic traits: practical considerations. *Nat. Rev. Genet.* 3 391–397. 10.1038/nrg796 11988764

[B88] Thimm O. Bläsing O. Gibon Y. Nagel A. Meyer S. Krüger P. (2004). Mapman: a user-driven tool to display genomics data sets onto diagrams of metabolic pathways and other biological processes. *Plant J.* 37 914. 10.1111/j.1365-313X.2004.02016.x 14996223

[B89] Udall J. A. Quijada P. A. Lambert B. Osborn T. C. (2006). Quantitative trait analysis of seed yield and other complex traits in hybrid spring rapeseed (*Brassica napus* L.): 2. Identification of alleles from unadapted germplasm. *Theor. Appl. Genet.* 113 597–609. 10.1007/s00122-006-0324-0 16767446

[B90] Ulitsky I. Shlomi T. Kupiec M. Shamir R. (2008). From E-MAPs to module maps: dissecting quantitative genetic interactions using physical interactions. *Mol. Syst. Biol.* 4:209. 10.1038/msb.2008.42 18628749 PMC2516364

[B91] Wang F. Guan C. Y. (2010). Molecular mapping and identification of quantitative trait loci for yield components in rapeseed (*Brasscia napus* L.). *Yi Chuan (Hereditas)*. 32 271–277. 10.3724/SP.J.1005.2010.00271 20233705

[B92] Wang N. Chen B. Xu K. Gao G. Li F. Qiao J. (2016). Association mapping of flowering time QTLs and insight into their contributions to rapeseed growth habits. *Front. Plant Sci.* 24:338. 10.3389/fpls.2016.00338 27047517 PMC4805649

[B93] Wang X. Chen L. Wang A. Wang H. Tian J. Zhao X. (2016). Quantitative trait loci analysis and genome-wide comparison for silique related traits in *Brassica napus*. *BMC Plant Biol.* 16:71. 10.1186/s12870-016-0759-7 27000872 PMC4802616

[B94] Wang X. Wang H. Long Y. Li D. Yin Y. Tian J. (2013). Identification of QTLs associated with oil content in a high-oil *Brassica napus* cultivar and construction of a high-density consensus map for QTLs comparison in *B. napus*. *PLoS One* 8:e80569. 10.1371/journal.pone.0080569 24312482 PMC3846612

[B95] Wigge P. A. Kim M. C. Jaeger K. E. Busch W. Schmid M. Lohmann J. U. (2005). Integration of spatial and temporal information during floral induction in *Arabidopsis*. *Science* 309 1056–1059. 10.1126/science.1114358 16099980

[B96] Yamaguchi S. (2008). Gibberellin metabolism and its regulation. *Annu. Rev. Plant Biol.* 59 225–251. 10.1146/annurev.arplant.59.032607.092804 18173378

[B97] Yang P. Shu C. Chen L. Xu J. Wu J. Liu K. (2012). Identification of a major QTL for silique length and seed weight in oilseed rape (*Brassica napus* L.). *Theor. Appl. Genet.* 125 285–296. 10.1007/s00122-012-1833-7 22406980

[B98] Yang Y. Shi J. Wang X. Liu G. Wang H. (2016). Genetic architecture and mechanism of seed number per pod in rapeseed: elucidated through linkage and near-isogenic line analysis. *Sci. Rep.* 6:24124. 10.1038/srep24124 27067010 PMC4828700

[B99] Ye J. Yang Y. Chen B. Shi J. Luo M. Zhan J. (2017). An integrated analysis of QTL mapping and RNA sequencing provides further insights and promising candidates for pod number variation in rapeseed (*Brassica napus* L.). *BMC Genomics* 18:71. 10.1186/s12864-016-3402-y 28077071 PMC5225578

[B100] Yuan H. M. Huang X. (2016). Inhibition of root meristem growth by cadmium involves nitric oxide-mediated repression of auxin accumulation and signalling in *Arabidopsis*. *Plant Cell Environ.* 39 120–135. 10.1111/pce.12597 26138870

[B101] Zeevaart J. A. D. (2008). Leaf-produced floral signals. *Curr. Opin. Plant Biol.* 11 541–547. 10.1016/j.pbi.2008.06.009 18691931

[B102] Zhang G. Zhou W. (2006). Genetic analyses of agronomic and seed quality traits of synthetic oilseed *Brassica napus* produced from interspecific hybridization of *B. campestris and B. oleracea*. *J. Genet.* 85 45–51. 10.1007/BF02728969 16809839

[B103] Zhang L. Li S. Chen L. Yang G. (2012). Identification and mapping of a major dominant quantitative trait locus controlling seeds per silique as a single Mendelian factor in *Brassica napus L. Theor. Appl. Genet.* 125 695–705. 10.1007/s00122-012-1861-3 22487878

[B104] Zhang L. Yang G. Liu P. Hong D. Li S. He Q. (2011). Genetic and correlation analysis of silique-traits in *Brassica napus* L. by quantitative trait locus mapping. *Theor. Appl. Genet.* 122 21–31. 10.1007/s00122-010-1419-1 20686746

[B105] Zhang S. Li C. Zhou Y. Wang X. Li H. Feng Z. (2018). TANDEM ZINC-FINGER/PLUS3 is a key component of phytochrome a signaling. *Plant Cell* 30 835–852. 10.1105/tpc.17.00677 29588390 PMC5973844

[B106] Zhang Y. Liu Z. Wang X. Wang J. Fan K. Li Z. (2018). DELLA proteins negatively regulate dark-induced senescence and chlorophyll degradation in *Arabidopsis* through interaction with the transcription factor WRKY6. *Plant Cell Rep.* 37 981–992. 10.1007/s00299-018-2282-9 29574486

[B107] Zhao J. Becker H. C. Zhang D. Ecke W. Zhang Y. (2005). Oil content in a European × Chinese rapeseed population: QTL with additive and epistatic effects and their genotype-environment interactions. *Crop Sci.* 45, 51–59.

[B108] Zhao W. Wang X. Wang H. Tian J. Li B. Chen L. (2016). Genome-wide identification of QTL for seed yield and yield-related traits and construction of a high-density consensus map for QTL comparison in *Brassica napus*. *Front. Plant Sci.* 7:17. 10.3389/fpls.2016.00017 26858737 PMC4729939

[B109] Zheng M. Peng C. Liu H. Tang M. Yang H. Li X. (2017). Genome-wide association study reveals candidate genes for control of plant height, branch initiation height and branch number in rapeseed (*Brassica napus* L.). *Front. Plant Sci.* 8:1246. 10.3389/fpls.2017.01246 28769955 PMC5513965

[B110] Zhou J. Liu D. Wang P. Ma X. Lin W. Chen S. (2018). Regulation of *Arabidopsis* brassinosteroid receptor BRI1 endocytosis and degradation by plant U-box PUB12/PUB13-mediated ubiquitination. *Proc. Natl. Acad. Sci. U.S.A.* 115 E1906–E1915. 10.1073/pnas.1712251115 29432171 PMC5828578

[B111] Zhou Q. H. Fu D. H. Mason A. S. Zeng Y. J. Zhao C. X. Huang Y. J. (2014). In silico integration of quantitative trait loci for seed yield and yield-related traits in *Brassica*. *Mol. Breed.* 33 881–894. 10.1007/s11032-013-0002-2

[B112] Zhu M. Zhao S. (2007). Candidate gene identification approach: progress and challenges. *Int. J. Biol. Sci.* 3 420–427. 10.7150/ijbs.3.420 17998950 PMC2043166

